# Using Zhang’s supertension-relieving suture technique with slowly-absorbable barbed sutures in the management of pathological scars: a multicenter retrospective study

**DOI:** 10.1093/burnst/tkad026

**Published:** 2023-06-15

**Authors:** Peiru Min, Shunuo Zhang, Dorsa Gholamali Sinaki, Ping Yao, Fuhua Hu, Xin Wang, Danya Zhou, Jun Chai, Yixin Zhang

**Affiliations:** Department of Plastic and Reconstructive Surgery, Shanghai Ninth People's Hospital Affiliated to Shanghai Jiao Tong University School of Medicine, 639 ZhiZaoJu Road, Huangpu District, Shanghai, 200011, China; Department of Plastic and Reconstructive Surgery, Shanghai Ninth People's Hospital Affiliated to Shanghai Jiao Tong University School of Medicine, 639 ZhiZaoJu Road, Huangpu District, Shanghai, 200011, China; Department of Plastic and Reconstructive Surgery, Shanghai Ninth People's Hospital Affiliated to Shanghai Jiao Tong University School of Medicine, 639 ZhiZaoJu Road, Huangpu District, Shanghai, 200011, China; Department of Plastic Surgery, Hangzhou Plastic Surgery Hospital, 168 Shangtang Road, Xiacheng District, Hangzhou, 310000, Zhejiang, China; Department of Plastic Surgery, Hangzhou Plastic Surgery Hospital, 168 Shangtang Road, Xiacheng District, Hangzhou, 310000, Zhejiang, China; Department of Plastic and Reconstructive Surgery, Ningbo Sixth Hospital, 1059 East Zhongshan Road, Yinzhou District, Ningbo, 315040, Zhejiang, China; Department of Plastic and Reconstructive Surgery, Ningbo Sixth Hospital, 1059 East Zhongshan Road, Yinzhou District, Ningbo, 315040, Zhejiang, China; Department of Plastic and Reconstructive Surgery, Shanghai Ninth People's Hospital Affiliated to Shanghai Jiao Tong University School of Medicine, 639 ZhiZaoJu Road, Huangpu District, Shanghai, 200011, China; Department of Plastic and Reconstructive Surgery, Shanghai Ninth People's Hospital Affiliated to Shanghai Jiao Tong University School of Medicine, 639 ZhiZaoJu Road, Huangpu District, Shanghai, 200011, China; Department of Burns and Plastic Surgery, Ruijin Hospital Affiliated to Shanghai Jiao Tong University School of Medicine, 197 RuiJinEr Road, Huangpu District, Shanghai, 200020, China

**Keywords:** Pathological scar, Tension-relieving, Suture technique, Tension

## Abstract

**Background:**

An ideal tension-relieving suture should be efficient for >3 months to retrieve normal tensile strength. Most preexisting suturing techniques provided tension elimination followed by relapse and scar proliferation due to absorption and cut-through of the sutures. This study introduces a simple but effective suture technique developed by a senior author (ZYX) to solve this problem.

**Methods:**

A total of 120 patients with pathological scar (PS) had intervention treatment with the proposed suturing strategy at three centers from January 2018 to January 2021. A slowly absorbable 2–0 barbed suture was used for subcutaneous tension relieving with a set-back from the wound edge and a horizontal interval between proposed inserting points of 1 cm. The Patient and Observer Scar Assessment Scale (POSAS), scar width, perfusion and eversion of the wound edge were evaluated at 3-, 6- and 12-month follow-up. The time needed to place the tension-relieving suture was recorded and relapse was monitored for 18 months postoperatively.

**Results:**

In total, 76 trunks, 32 extremities and 12 cervical PS were included, with an average subcutaneous tension-relieving suture time of 5 min. The Patient and Observer Scar Assessment Scale (POSAS) score decreased from 84.70 ± 7.06 preoperatively to 28.83 ± 3.09, 26.14 ± 1.92 and 24.71 ± 2.00 at 3, 6 and 12 months postoperatively, respectively (*p* < 0.0001). The scar widths were 0.17 ± 0.08, 0.25 ± 0.09 and 0.33 ± 0.10 cm, respectively, with perfusion significantly decreased from 213.64 ± 14.97 to 112.23 ± 8.18 at 6 months (*p* < 0.0001). The wound edge flattened out during the first 3 months in most cases with only two scar relapses.

**Conclusions:**

Zhang’s suture technique provides a rapid and long-lasting tension-relieving effect with ideal scar appearances and lower relapse rates in the surgical management of PS.

## Background

Human skin exhibits significant resting mechanical forces ranging from 0.40 to 0.98 N, which are larger than any other species, with the stress increasing ~2-fold during wound healing [1]. Although wounds undergo persistent tension for a period of up to 1 year, injured skin tissue requires ~3 months to progressively retrieve 80% of normal tensile strength while gaining most of its stress within the first 3 months under the impact of both internal and external forces [2–5]. Passive tension, as well as active cellular tension of collagen fibrils, constitute the internal forces in the human dermis [6, 7], and external forces caused by physical activities increase the tension in the human dermis by activating interactions between fibroblasts [8].

Pathological scar (PS), including keloid and hypertrophic scar (HS), is an abnormal type of wound healing that leads to functional and aesthetic impairment. Mechanical forces not only promote PS growth but also act as a primary trigger for their generation and recurrence [9]. As natural mechanical properties differ by body region, areas of greater mechanical stimulation show an increased incidence of PS formation [10]. Consequently, postoperative tension-relieving maneuvers are highly recommended, especially during the first 3 months after surgical intervention, for an optimized outcome as well as to avoid PS recurrence.

Suturing technique has assumed critical importance in interfering with wound healing and scar appearance [11, 12]. Different suturing strategies have evolved to achieve better surgical outcomes for wounds under tensions and all have their limitations [11]. The traditional transverse-mattress and vertical-mattress sutures gather up the epidermis and dermis together, but the tension-reduction effect may disappear once the stitches are removed [13, 14]. The modified buried vertical-mattress suture, the running-butterfly suture and the set-back suture have significant limitations, such as complex operative steps, prolonged operation time and risk of suture extrusion. More importantly, absorbable sutures cannot efficiently provide a long-lasting tension-relieving effect for >3 months [13–16]. In 2014, Wang *et al*. described a supertension-reduction suture for chest keloids [17]. Despite an improved outcome, with only 2.2% relapse, intradermal braided silk sutures tend to cut-through the tissue under tension, followed by a long-term risk of foreign-body reaction. Moreover, interrupted suturing with the braiding maneuver significantly prolonged the operation time.

To achieve an ideal suture technique in terms of long-lasting tension reduction, precise wound-edge approximation, low rate of suture extrusion and simple surgical procedure, we applied the slowly absorbable (180 days) barbed suture with our specific running suturing technique in PS patients at three institutes. Taking advantage of the biomechanical characteristics of the suture material, optimized surgical outcomes could be achieved. Here, we present our detailed experience in using Zhang’s supertension-relieving suture technique after PS excision under significant tension.

## Methods

### Patient selection

This was a multicenter retrospective study. Data of all 120 patients who underwent surgical excision of PS were obtained from the three hospitals in China between January 2018 and January 2021. The inclusion criteria embraced HS or keloids located on the trunk, extremities or cervical areas. A ‘pinch test’ was used to evaluate topical skin tension for the feasibility of primary closure. Patients with preexisting vascular or venous disease, neoplastic disease or oversized PS were excluded from this study. The present study was approved by the institutional review boards of Shanghai Ninth People’s Hospital (SH9H-2022-T299–1), Ningbo Sixth Hospital (YLYLS2022144) and Hangzhou Plastic Surgery Hospital (HZLL20210011).

### Surgical technique

**Figure 1:**
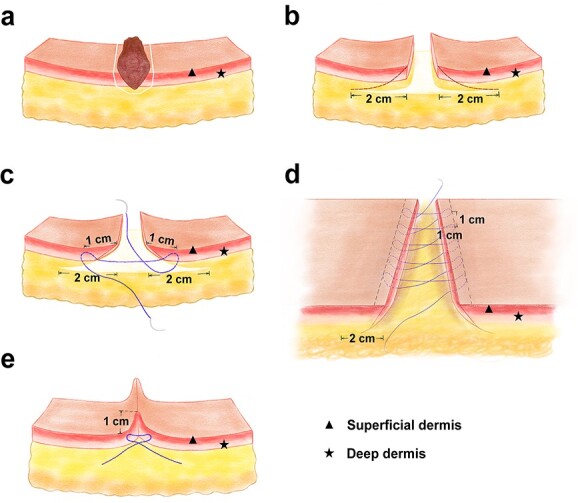
Schematic drawing of Zhang’s supertension-relieving suture. (**a**) Wedge-shaped scar excision, (**b**) enlarged undermining territory before (**c**, **d**) and after (**e**) knotting

All operations were performed under general or local anesthesia. After landmark along the edges of the scar with methylene blue, full-thickness excision was performed (Figure 1a). An undermining territory 2 cm away from the wound edge was then carefully prepared at the superficial fascia layer, with visible perforators preserved and redundant subcutaneous adipose tissue removed for better vascularization (Figure 1b). According to our experiences, large fatty tissues should be removed, whereas compact and small ones should be preserved for flap safety. Wedge-shaped excision was then employed as described by Zhang (Figure 1c) [18]. Hemostasis was carefully performed.

After temporary closure with #1 braided silk sutures, two parallel landmarks were made at a distance of 10 mm from the closed wound edge. The temporarily-closed wound was evenly distributed with an interval of 10 mm which represented the supposed inserting points (Figure 1d). A 2–0 bidirectional knotless suture (Spiral PDO STRATAFIX TM; Ethicon, Inc., USA) was used for subcutaneous tension-relieving purposes. The running suture was started from the midpoint, with one needle inserting a deep subcutaneous layer to the superficial dermis in a buried set-back fashion. After catching 2–3 mm of dermal tissue for a robust stress loading, the needle was angled down out of the subcutaneous tissue. Mirroring sutures were then applied and tightened at the proposed inserting points one by one for half of the wound. The needle was then turned around and travelled back superficially in the deep dermis layer towards the midline, with the barbs locking to prevent loosening of the wound. The remaining half of the wound was subcutaneously closed in a similar manner (Figure 1e). When the two needles met at the midline, one knot was tied and buried subcutaneously. The time required to perform the tension-relieving suture technique was recorded for each patient.

Finally, we performed an interrupted buried vertical-mattress suture as described by Zitelli and Moy [14] for mid-dermis layer closure with 5–0 PDS suture (PDS II^®^; Ethicon, Inc., USA), followed by routine out-layer suture to achieve excellent wound edge approximations. The options can be, but are not restricted to, 5–0 PDS suture or 4–0 Ti-Ni memory alloy wire (FULLTAI^®^; Shaanxi Futai Medical Technology Co Ltd, China). Based on our experiences, the out-layer suture can be removed 7 days postoperatively for trunk and 10 days postoperatively for extremities without any wound tension. Silicone sheets were used for 6 months, and the patient was instructed to avoid strenuous activity that affected the surgical site for 12 months postoperatively.

### Fractionated radiation therapy

Patients aged >16 years with a diagnosis of keloids or intractable HS received postoperative fractionated radiation therapy [19, 20]. The radiation therapeutic strategy was determined by one skilled radiologist and radiotherapy was performed 1 cm outside the incision border at a source-to-skin distance of 100 cm using Synergy (Elekta™). A total dose of 18 Gy for 3 consecutive days was administered within 24 h after surgery for all our patients as recommended [21–24]. In this case, a biologically effective dose of at least 30–40 Gy could be achieved to keep the keloid recurrence rate at <10% [25]. For patients with intractable HS located in high-tension sites, an empirical strategy with 12 Gy on 2 consecutive days was carried out for prophylactic treatment.

### Outcome assessment

The Patient and Observer Scar Assessment Scale (POSAS) included the Patient Scar Assessment Scale (PSAS) and the Observer Scar Assessment Scale (OSAS), which were used to evaluate the surgical improvement at 3-, 6- and 12-month follow-up. The PSAS includes six items: pain, pruritus, color, stiffness, thickness and irregularity, while the 6 items included in OSAS are: vascularity, pigmentation, thickness, relief, pliability, and surface. Each item was graded on a 10-point scale (1 represented the best and 10 represented the worst), and the scores on all items were summed to obtain a total score ranging from 6 to 60 [26]. Recurrence of PS was defined as a POSAS thickness score$>$5. The aspect and scar width were individually monitored for 18 months [27]. Topical perfusion was measured with the PeriCam PSI System (Perimed, Sweden) using the Region of Interests (ROIs) method.

Wound eversion was objectively assessed with a ruler on the adjacent flat skin surface parallelly aside from the midpoint. Data were collected immediately after surgery and at 3, 6 and 12 months postoperatively. Complications such as wound dehiscence, seroma or hematoma, infection and spitting sutures were observed and recorded for at least 12 months after surgery.

### Statistical analyses

Categorical variables are presented as numbers and percentages, and continuous data are expressed as mean ± standard deviation (SD), unless otherwise specified. Analyses were conducted using PROC GLM in SAS statistical software, version 9.4 (SAS Institute, Inc.). One-way within-group Analysis of Variance (ANOVA) test was applied to analyze each outcome (total POSAS score, PSAS score, OSAS score and wound eversion) at different time points. If significant, *post hoc* pairwise Tukey test was applied to compare differences between time points. We constructed four generalized linear models with random-effect intercepts for each subject to estimate the effects of our supertension-relieving suture technique on each outcome (total POSAS score, PSAS score, OSAS score and wound eversion), accounting for the repeated measures for each participant. We considered all statistical tests with *p* values <0.05 to be significant.

## Results

### Patient characteristics

Our suture technique was applied to 120 patients aged from 14 to 65 years, with an average age of 34 years (Table 1). Based on pathological sections, 80 patients were diagnosed with keloids and 40 with HS. The positions involved included the trunk (76 cases), the extremities (32 cases) and the cervical area (12 cases). PS dimensions ranged from 4 × 2 to 25 × 4 cm.

**Table 1:** Patient characteristics

**Patient characteristics**	**Value (%)**
No. of patients	120 (50% from Shanghai 9^th^ People’s Hospital, 25% each from Ningbo 6^th^ Hospital and Hangzhou Plastic Surgery Hospital)
Age (years)	
Mean	34
Range	14–65
Sex	
Male	73 (60.8)
Female	47 (39.2)
Scar type	
Hypertrophic scar	80 (66.7)
Keloid	40 (33.3)
Scar relapse	
Hypertrophic scar	0
Keloid	2 (1.7)
Scar position	
Trunk	76 (63.3)
Extremities	32 (26.7)
Cervical area	12 (10.0)
Scar dimension (cm)	
Range	4 × 2–25 × 4
Subcutaneous tension-relieving suture time (min)	
Mean ± SD	5.25 ± 1.36

All patients achieved primary closure without major complications. Among all patients from the three institutes, relapse was observed in only two cases (1.7%) after 18 months. The average time required to place the subcutaneous tension-relieving suture was 5.25 ± 1.36 min. After radiation therapy, 24 patients (20.0%) developed mild skin reactions of temporary pigmentation and slight squamous epithelial desquamation in the irradiated area; both of these reactions eventually disappeared.

### POSAS scores

**Figure 2:**
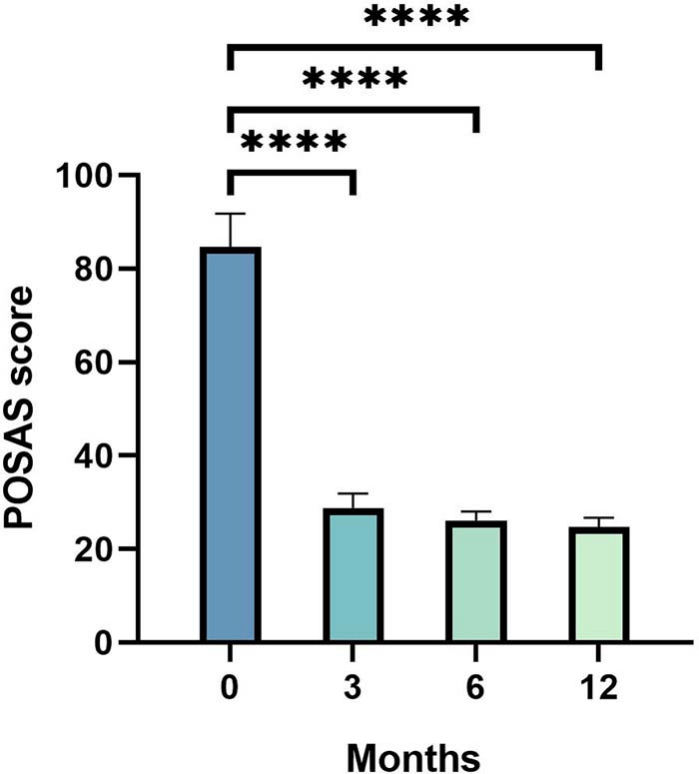
POSAS scores evaluated before surgery and at the 3-, 6-and 12-month follow-up after surgery; ^*^^*^^*^^*^p < 0.0001. *POSAS* Patient and Observer Scar Assessment Scale

**Figure 3:**
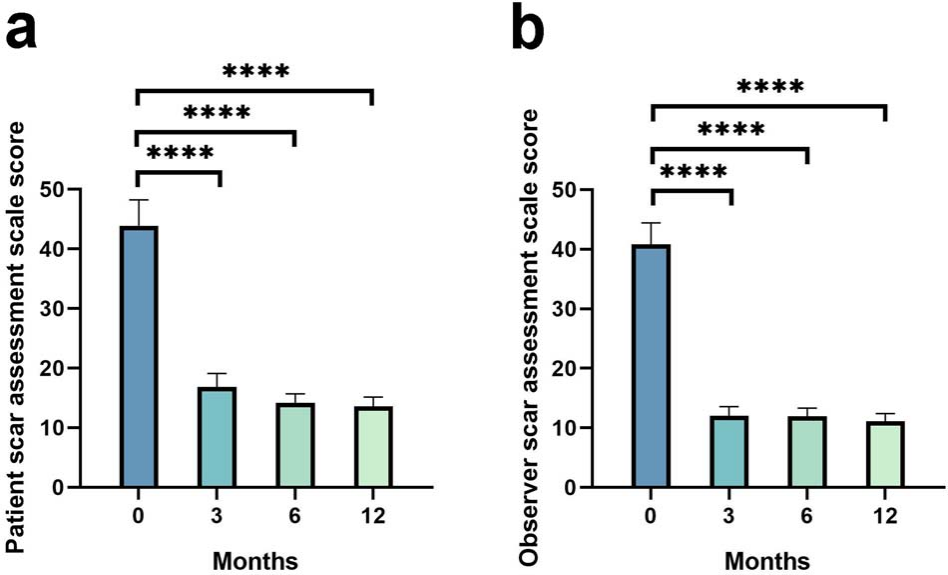
PSAS score (**a**) and OSAS score (**b**) evaluated before surgery and at 3-, 6-and 12-month follow-up after surgery; ^*^^*^^*^^*^p < 0.0001. *PSAS* Patient Scar Assessment Scale, *OSAS *Observer Scar Assessment Scale Assessment Scale

The total mean POSAS score decreased from 84.70 ± 7.06 preoperatively to 28.83 ± 3.09, 26.14 ± 1.92 and 24.71 ± 2.00 at the 3-, 6- and 12-month follow-up, respectively, which indicates significant improvement after performing Zhang’s supertension-relieving suture technique (*p* < 0.0001) (Figure 2, Table 2). The mean PSAS and OSAS scores were 43.91 ± 4.31, 16.83 ± 2.28, 14.18 ± 1.50, 13.62 ± 1.55 and 40.79 ± 3.64, 12.00 ± 1.55, 11.96 ± 1.37, 11.09 ± 1.28 at baseline and at the 3-, 6- and 12-month follow-up, respectively, indicating significant differences (*p* < 0.0001) (Figure 3, Table 2). Meanwhile, a significant improvement was observed in POSAS subgroups including pain, pruritus, color, stiffness, vascularity, pigmentation, relief, pliability and surface at the 12-month follow-up when compared to the baseline, whereas a slight relapse between 3 and 12 postoperative months in scar thickness and irregularity strongly indicated the absorption of the tension-relieving suture (Figure S1, Figure S2, see online supplementary material).

**Table 2:** Effects of our supertension-relieving suture technique on each outcome based on a generalized linear model

**Outcomes**	**Effect**	***F* value**	**Pr > *F***	**Comparison**		***F* value**	**Adjusted *p* value**
				**Time (months)**	**Time (months)**		
POSAS	Time	6351.43	<0.0001	0	3	6635.15	<0.0001
				0	6	7924.03	<0.0001
				0	12	8352.64	<0.0001
				3	6	63.24	<0.0001
				3	12	151.98	<0.0001
				6	12	48.14	<0.0001
PSAS	Time	3613.29	<0.0001	0	3	3457.87	<0.0001
				0	6	5079.65	<0.0001
				0	12	5073.24	<0.0001
				3	6	125.57	<0.0001
				3	12	182.42	<0.0001
				6	12	11.71	0.0009
OSAS	Time	5844.01	<0.0001	0	3	7399.43	<0.0001
				0	6	6789.08	<0.0001
				0	12	8302.30	<0.0001
				3	6	0.05	0.8253
				3	12	23.28	<0.0001
				6	12	31.75	<0.0001
Wound eversion	Time	1672.89	<0.0001	0	3	1397.51	<0.0001
				0	6	1871.22	<0.0001
				0	12	2181.25	<0.0001
				3	6	357.18	<0.0001
				3	12	597.77	<0.0001
				6	12	192.71	<0.0001

The *F* value and Pr > *F* in table 2 represent the result of model construction. The model we constructed in our study is deemed to be meaningful when Pr > *F* < 0.05. The right two columns of table 2 are *F* value and adjusted *p* value. The adjusted *p* value <0.05 suggests the difference between different time points are statistically significant. *POSAS* Patient and Observer Scar Assessment Scale, *PSAS* Patient Scar Assessment Scale, *OSAS* Observer Scar Assessment Scale

### Scar width

**Figure 4:**
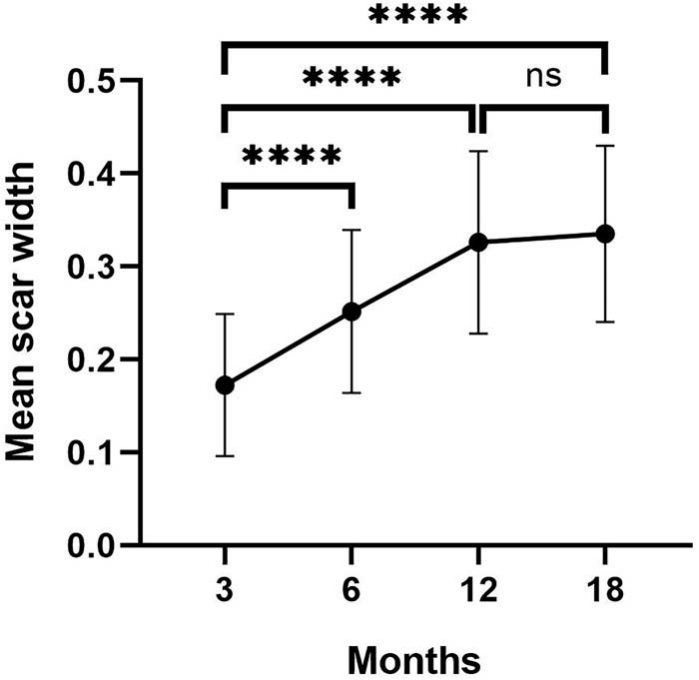
Mean scar width at 3-, 6-, 12- and 18-month follow-up postoperatively; ^*^^*^^*^^*^p < 0.0001, ns indicates no significance

The mean scar width was 0.17 ± 0.08, 0.25 ± 0.09, 0.33 ± 0.10 and 0.34 ± 0.09 cm at 3-, 6-, 12- and 18-month follow-up, respectively. The results showed significant differences between 3 and 6, 3 and 12, and 3 and 18 months. However, the scar width no longer increased after 12 months postoperatively (*p* = 0.8588), indicating maturity and stabilization of the surgical scar (Figure 4).

### Wound perfusion

**Figure 5:**
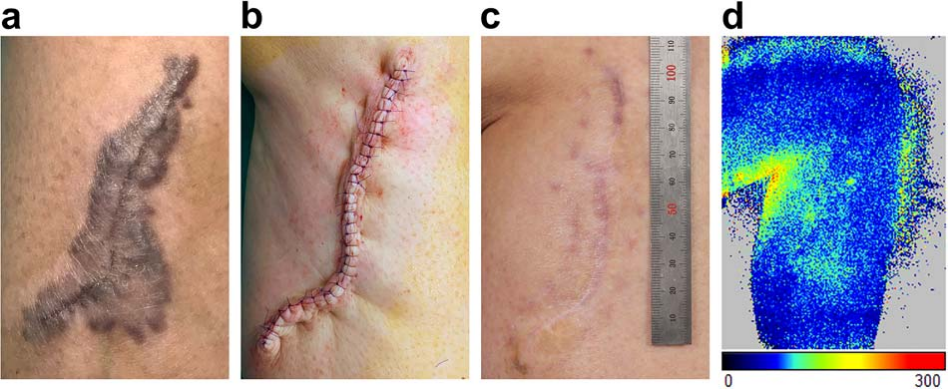
Photographs of a 26-year-old female patient with right leg hypertrophic scar. (**a**) Preoperative, (**b**) immediately after surgery, (**c**) 6 months postoperative, and (**d**) wound blood flow 6 months postoperative

After the surgery, the mean ROI decreased to 178.26 ± 10.52 and 112.23 ± 8.18 at 1- and 6-month follow-up, respectively (both *p* < 0.0001 compared with 213.64 ± 14.97 preoperatively). The results showed that Zhang’s supertension-relieving suture technique significantly reduced local wound perfusion (Figure 5).

### Eversion of the wound edge

**Figure 6:**
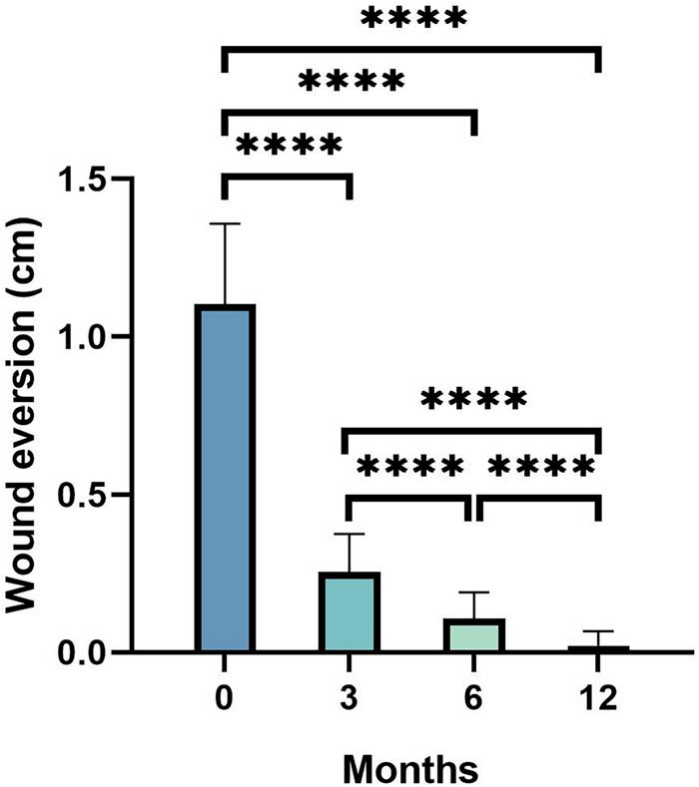
Eversion of wound edge immediately after surgery and at 3-, 6-, 12-month follow-up postoperatively; ^*^^*^^*^^*^p < 0.0001

The over-everted hump at the incision began to flatten at 1 month post-operation and was flattened in 96.7% of cases after 3 months. The mean postoperative wound edge eversion height was 1.10 ± 0.26 cm immediately after the operation, and it was reduced to 0.26 ± 0.12, 0.11 ± 0.08 and 0.02 ± 0.04 cm at 3-, 6-, and 12-month follow-up, respectively (all *p* < 0.0001) (Figure 6, Table 2). The wound edge remained everted in 6 patients at 12-month follow-up. Thus, active stretching exercises were asked to be carried out at the surgical site, which was followed by complete flattening within an additional 2 months.

### Typical cases

#### Clinical case 1

**Figure 7:**
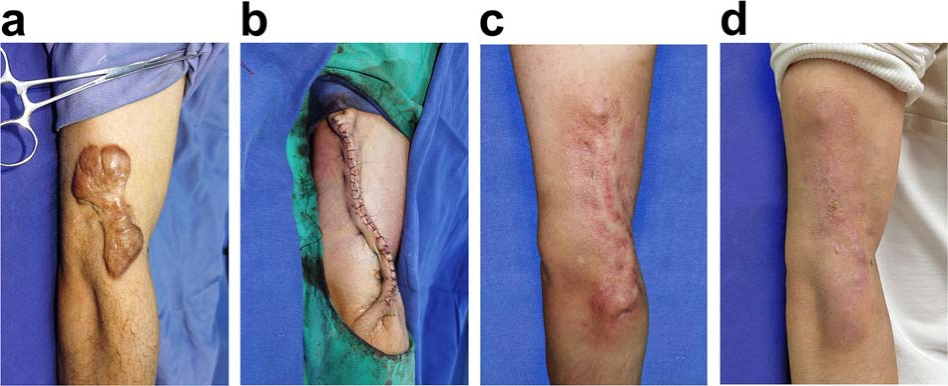
Photographs of a 28-year-old male patient with right upper limb keloid. Radiotherapy was applied within 24 h postoperatively. (**a**) Preoperative, (**b**) immediately after the surgery, (**c**) 3 months postoperative, and (**d**) 18 months postoperative

A 28-year-old male patient presented with a 4-year history of keloid measuring 10 × 5 cm in the right upper limb (Figure 7a). Surgical excision of the keloid was performed after standard sterilization. Once undermining was carefully performed at the superficial fascia layer, Zhang’s supertension-relieving suturing was carried out with 5–0 PDS for out-layer closure (Figure 7b). This patient received radiotherapy within 24 h after surgery. At the 3-month follow-up, the everted wound edge was observed to be somewhat flattened (Figure 7c). The surgical outcome was excellent at 18 months postoperatively (Figure 7d).

#### Clinical case 2

**Figure 8:**
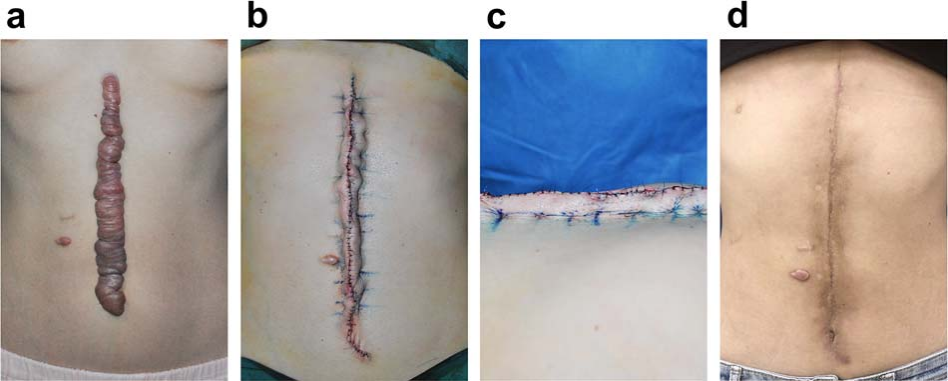
Photographs of a 32-year-old female patient with chest/abdomen keloid. Radiotherapy was applied within 24 h postoperatively. (**a**) Preoperative. (**b**) immediately after surgery, (**c**) lateral view of over-everted wound edge, and (**d**) 18 months postoperative

A 32-year-old female suffered from an extremely large keloid measuring 25 × 4 cm in the chest/abdominal area (Figure 8a). We performed Zhang’s supertension-relieving suture after scar excision (Figure 8b). The wound skin was everted with a height of 10 mm immediately after the surgery, followed by radiotherapy within 24 h postoperatively (Figure 8c). At the 12-month follow-up, an ideal appearance was observed at the surgical site, with the everted skin flattened with slight pigmentation in the irradiated area (Figure 8d).

#### Clinical case 3

**Figure 9:**
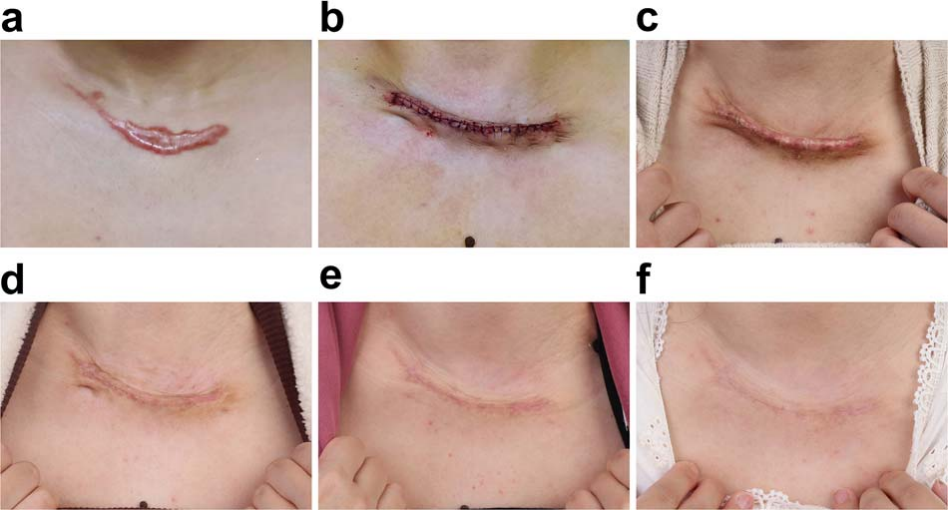
Photographs of a 35-year-old female patient with cervical hypertrophic scar. (**a**) Preoperative, (**b**) immediately after surgery and (**c**–**f**) 1, 3, 6 and 18 months postoperative, respectively

A 35-year-old female with an 8 × 2.5 cm cervical HS underwent scar excision in our institution (Figure 9a). The patient received our technique without postoperative radiotherapy (Figure 9b). The wound skin remained everted at the 1-month follow-up (Figure 9c) and began to flatten at 2 months postoperatively (Figure 9d). At the 3-month follow-up, the skin was almost flattened (Figure 9e). At the 18-month follow-up, the patient was satisfied with the surgical outcome (Figure 9f).

## Discussion

PSs characterized by histological Extracellular Matrix (ECM) accumulation are notorious for their clinical invasiveness, associated irritating symptoms and topical contracture. As a multifactorial disease, persistent mechanical tension during wound healing was found to be crucial to scar formation [28, 29]. Several studies have explored the potential mechanisms of mechanical tension in scar proliferation. Wong *et al*. [30] indicated that intracellular pathways convert tension into biochemical responses that lead to fibroblast proliferation. By mediating the transformation of fibroblasts into myofibroblasts, mechanical forces upregulate the synthesis and deposition of collagen, followed by the proliferation of PS [6].

Although relative mechanical tension may exist for up to 1 year, most unbeneficial wound tension appears within the first 3 months [2, 3]. Clinically, PS occurs more frequently in regions under significant tension such as the chest, scapula, and extremities. Surgical excision remains as the most straightforward management strategy; however, analysis of therapeutic outcomes showed a correlated higher recurrence rate at operative sites under tension [31, 32]. As stress across the incision tends to upregulate collagen deposition, thus leading to PS proliferation, effective tension-relieving is considered of great importance, especially during the first 3 months after PS excision [33]. Therefore, numerous modulations have been developed to eliminate wound tension, such as design along tension lines, proper patient positioning, skin-stretching techniques, pre-suturing and the use of various types of offload devices [34].

As the very last step of most traditional surgical procedures, suture techniques are critically important in the prevention of scar hyperplasia. To date, a number of different suture techniques have been developed for tension-relieving purposes and all have their limitations. Transverse-mattress and vertical-mattress sutures effectively reduce the tension across the wound only during the period prior to the removal of stitches. Liu *et al*. [35] described a modified intradermal buried vertical-mattress suture technique that posed a risk of suture extrusion to the epidermal surface because the superior arc of the suture is very close to the epidermis. The ‘super-tension-reduction’ suture was conducted without wedge-shaped excision, thus resulting in an enlarged wound tension, but the placement of multilayered interrupted sutures required a considerable amount of time during the surgical maneuver [17].

In this study, we describe Zhang’s supertension-relieving suture technique that has several key features. An undermining for about 2 cm away from both wound edges was routinely performed at the superficial fascia layer with wedge-shaped excisions. This dissection helped to take advantage of the viscoelastic property of skin tissue, thus reducing the wound tension and providing sufficient operating space for needle handling. Needle insertion was then performed at a distance of ~10 mm from both sides of the wound edge with a 10-mm interval to avoid causing skin ischemia. The deep, denser, septate fibrofatty lobules were identified with the superficial loose areolar fat and they were elevated together in a ‘super-thin flap’ fashion [36]. With this maneuver, the blood supply may be reliably ensured through indirect linking-vessel connections of the perforasome [37], while the relatively thinner skin paddle exhibited better flexibility for wound eversion under adequate tension. Redundant subcutaneous adipose tissue was carefully removed to avoid its impairment of wound approximation and angiogenesis of the overlying skin [38]. According to the results, wound perfusion decreased significantly from 213.64 ± 14.97 preoperatively to 112.23 ± 8.18 at the 6-month follow-up. Compared to the increased microvascular density of scar tissue, our technique may provide a relatively similar microenvironment to normal tissue oxygen levels during wound healing [39, 40].

Wound eversion should be conducted for tension-relieving purposes. Although Kappel [41] found no difference between the everted and planar sides, Wang [42] and Liu *et al*. [35] obtained superior cosmetic outcomes with wound eversion. The over-everted skin by our technique indicated effective tension-relief at the wound edge. Unlike other practitioners, we used slowly absorbable 2–0 bidirectional knotless sutures (Spiral PDO STRATAFIX TM; Ethicon, Inc., USA) for subcutaneous tension-relief. The barbs on the suture help to lock the tissue from loosening, with the wound tension evenly distributed to each insertion point of the needle without the interruption of tied knots [43]. The continuous suture maneuver provides a simple and time-saving method of deep-layer closure, thus not only reducing the total operation time but also the risk of infection.

A total of three suture layers were routinely carried out in our method. The deepest 2–0 subcutaneous suture plays a dominant role in tension reduction and wound eversion. The needle engaged sufficient dermal and subcutaneous tissue in the suture loop after knotting, thus providing reliable resistance against the retraction force on both sides. Based on our clinical observations, performing the technique completely subcutaneously may be infeasible, as the subcutaneous fascia and adipose tissues are too loose and fragile to provide robust and reliable control of stress loading, while the subcutaneous tissue is easily cut through under significant wound tension. It is worth noting that some anatomical areas with relatively thinner dermis showed potential risks of suture extrusion, in which case the 2–0 knotless suture should not be run too superficially to the superficial dermis. Thereafter, 5–0 PDS sutures were applied to perform a classic interrupted buried vertical-mattress with prolonged intervals in the mid-dermis layer for wound-edge approximation purposes. As a result, subcuticular closure is considered unnecessary and the out-layer was routinely closed for a regular cutaneous approximation. Taking into account the tremendous tension-relieving ability of our suture technique, the time to removal of the out-layer sutures could be anticipated to be 7 days for the trunk and 10 days for the extremities, without leaving any obvious marks on the skin due to foreign body reaction [11].

The change in POSAS from 24.71 ± 2.00 preoperatively to 84.70 ± 7.06 at 12 months indicated favorable outcomes after performing Zhang’s supertension-relieving suture technique among all 120 patients. In addition, the mean scar width at the 12-month follow-up was 0.33 ± 0.10 cm, indicating a better appearance compared to the previously reported 0.6–0.8 cm in high-tensional wounds [44]. We attribute this phenomenon to two factors. First, preservation of the perforators and removal of the redundant subcutaneous adipose tissue increases topical perfusion, thus promoting a hypoxic microenvironment during wound healing [45, 46]. Second, the elimination of wound tension effectively downregulates the proliferation and migration of inflammatory factors through the Transforming Growth Factor-β (TGF-β) pathway [29].

Although the 2–0 knotless suture is theoretically absorbed by ~180 days post-surgery, we believe that the mechanical stress affects the absorption rate [47]. The wound edges actually flattened out at nearly 3 months postoperatively, which indicated a critical period for the tension-relieving effect of our technique. Taking into account the importance of the first 3 months during HS and keloid formation [33], our modified suturing technique effectively eliminated wound tension and resulted in a relatively low relapse rate of 1.67% at the 18-month follow-up. Interestingly, abnormal eversions had not vanished in 6 out of 120 patients among all three institutes at the 12-month follow-up. Frequent strenuous activity in the surgical site was then requested and all the eversions got flattened after an additional 2 months.

To the best of our knowledge, Zhang’s supertension-relieving suture technique is particularly suitable for the management of PS on the trunk and extremities. However, this surgical strategy has limitations for facial areas because of the unsightly appearance. Moreover, skin tension and subcutaneous tissue thickness differ among individuals, anatomical sites and even sides of the body. Clinical experience with an understanding of skin perfusion is paramount. Last but not least, the importance of the suture technique vs. radiation therapy may not be statistically separated in the present study, but the benefit of our modified suture technique is undoubted. To shed light on this issue, a worldwide consensus on postoperative radiotherapy for PS with more prospective randomized controlled trials will be necessary.

## Conclusions

The present multicenter retrospective clinical study shows that Zhang’s supertension-relieving suture technique provides an effective and reliable option for high-tension wounds after surgical excision of PS in trunks and extremities. With our modified methods, excellent aesthetic and functional outcomes can be achieved within 6 months postoperatively, which significantly improves the patient’s quality of life. Therefore, Zhang’s supertension-relieving suture technique is a promising suture choice for Asians in the management of PS, especially at high-tension sites.

## Supplementary Material

SFig1_tkad026

SFig2_tkad026

## Data Availability

The data used in this publication are available upon request.

## Abbreviations

HS: Hypertrophic scar; OSAS: Observer Scar Assessment Scale; PS: Pathological scar; PSAS: Patient Scar Assessment Scale.

## Clinical trial registration

This trial was approved by the Ethics Committee of Shanghai Ninth People’s Hospital (registration number: SH9H-2022-T299–1), Ningbo Sixth Hospital (registration number: YLYLS2022144) and Hangzhou Plastic Surgery Hospital (registration number: HZLL20210011).
