# Supplementary figures and images for: Using Zhang’s supertension-relieving suture technique with slowly-absorbable barbed sutures in the management of pathological scars: a multicenter retrospective study
Source: Burns Trauma. 2023 Jun 15;11:tkad026. doi: 10.1093/burnst/tkad026 (PMC10271604; doi:10.1093/burnst/tkad026)

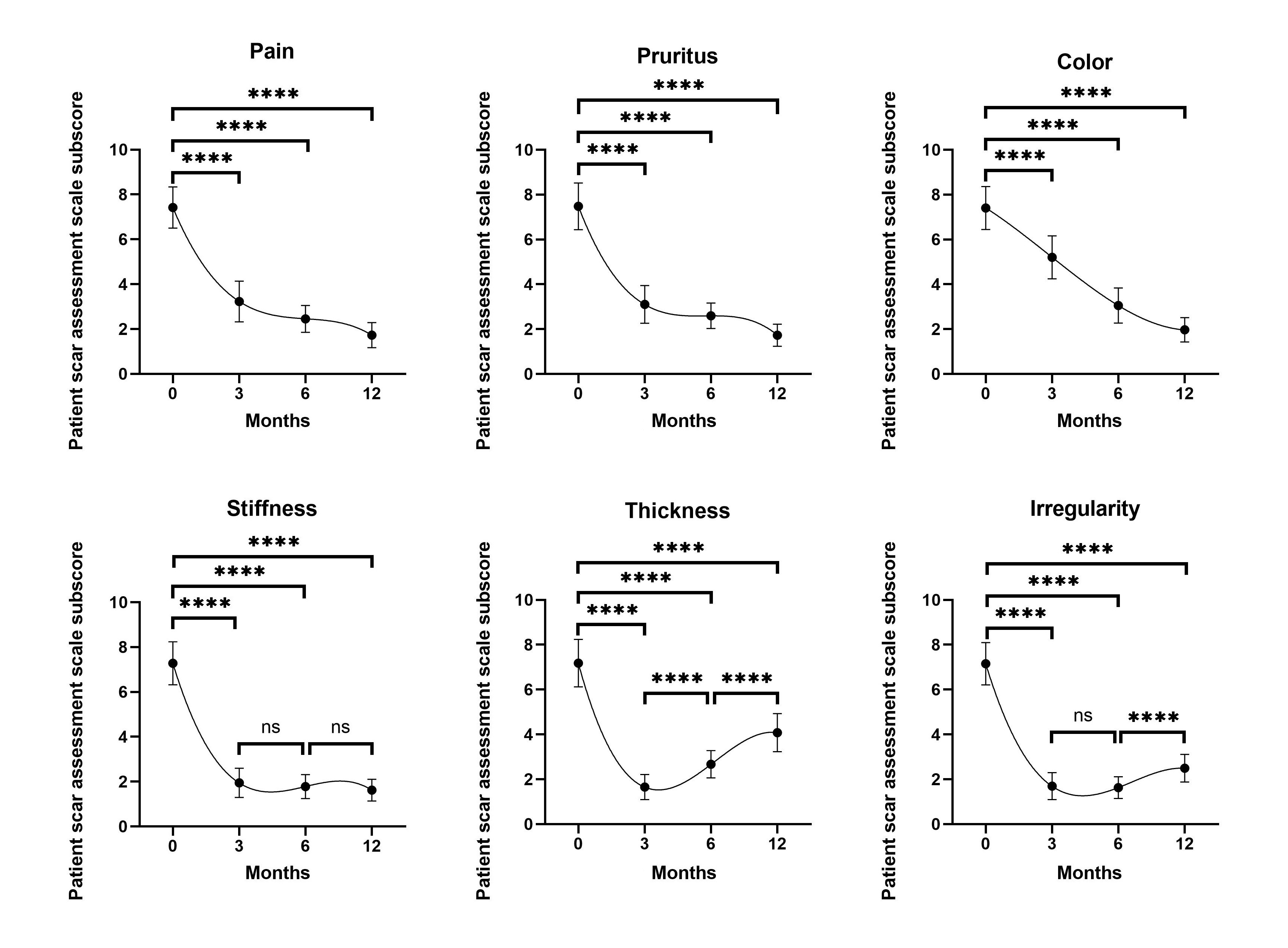

Supplement: SFig1_tkad026 [file sfig1_tkad026.jpeg]

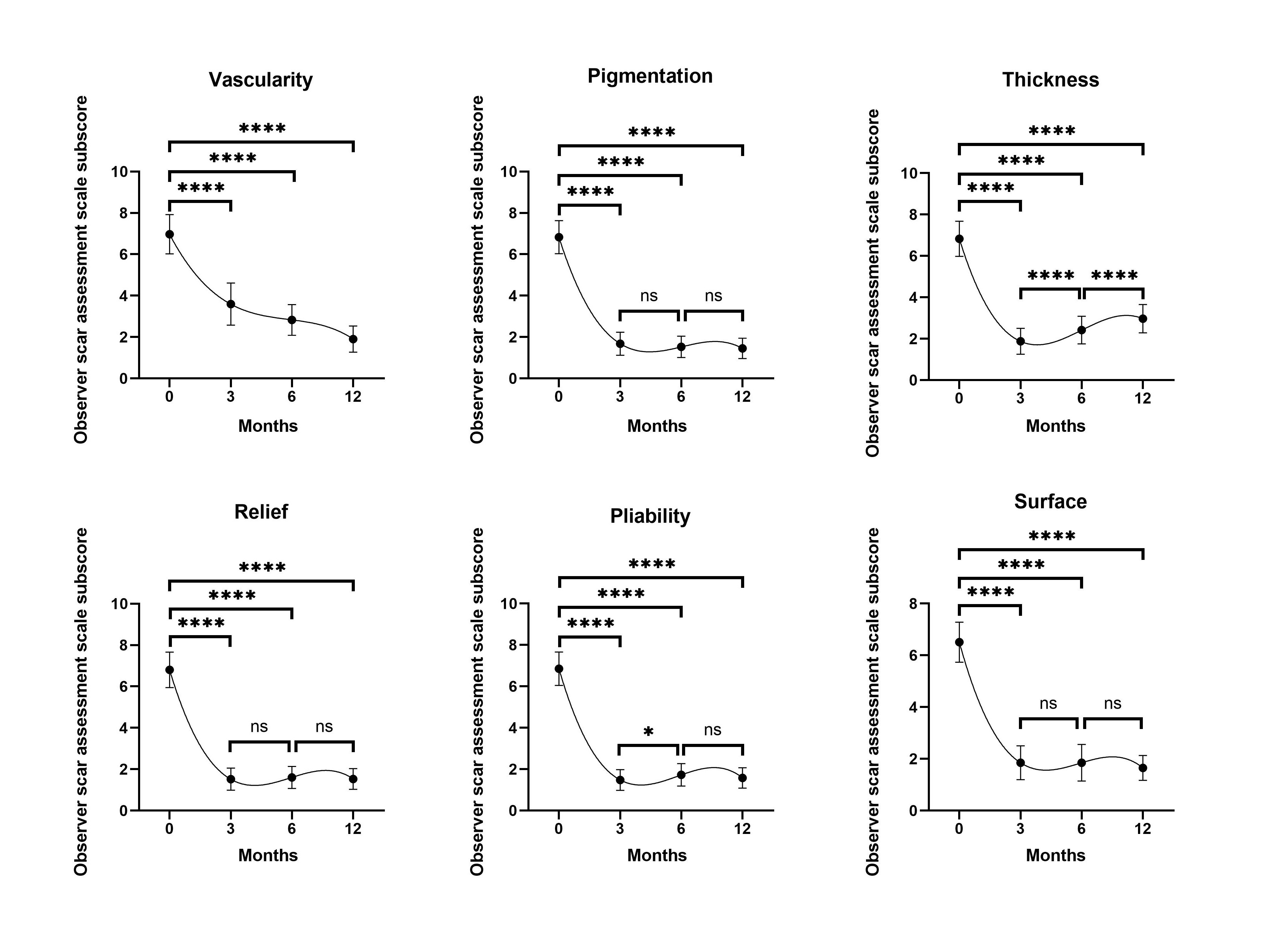

Supplement: SFig2_tkad026 [file sfig2_tkad026.jpeg]
